# 

**Published:** 1974-07

**Authors:** 


### BRISTOL MEDICO-CHIRURGICAL JOURNAL

SPECIAL NUMBER COMMEMORATING
THE CENTENARY
OF
THE BRISTOL MEDICO-CHIRURGICAL SOCIETY

JULY 1974

VOL. 89 (iii) No. 331

